# Role of Lipid Rafts and GM1 in the Segregation and Processing of Prion Protein

**DOI:** 10.1371/journal.pone.0098344

**Published:** 2014-05-23

**Authors:** Laura Botto, Diana Cunati, Silvia Coco, Silvia Sesana, Alessandra Bulbarelli, Emiliano Biasini, Laura Colombo, Alessandro Negro, Roberto Chiesa, Massimo Masserini, Paola Palestini

**Affiliations:** 1 Department of Health Science - Medical School, University of Milano-Bicocca, Monza, Italy; 2 Department of Neuroscience, IRCCS-Istituto di Ricerche Farmacologiche Mario Negri, Milano, Italy; 3 Department of Molecular Biochemistry and Pharmacology, IRCCS-Istituto di Ricerche Farmacologiche Mario Negri, Milano, Italy; 4 Department of Biomedical Sciences, University of Padova, Padova, Italy; INSERM, France

## Abstract

The prion protein (PrP^C^) is highly expressed within the nervous system. Similar to other GPI-anchored proteins, PrP^C^ is found in lipid rafts, membrane domains enriched in cholesterol and sphingolipids. PrP^C^ raft association, together with raft lipid composition, appears essential for the conversion of PrP^C^ into the scrapie isoform PrP^Sc,^ and the development of prion disease. Controversial findings were reported on the nature of PrP^C^-containing rafts, as well as on the distribution of PrP^C^ between rafts and non-raft membranes. We investigated PrP^C^/ganglioside relationships and their influence on PrP^C^ localization in a neuronal cellular model, cerebellar granule cells. Our findings argue that in these cells at least two PrP^C^ conformations coexist: in lipid rafts PrP^C^ is present in the native folding (α-helical), stabilized by chemico-physical condition, while it is mainly present in other membrane compartments in a PrP^Sc^-like conformation. We verified, by means of antibody reactivity and circular dichroism spectroscopy, that changes in lipid raft-ganglioside content alters PrP^C^ conformation and interaction with lipid bilayers, without modifying PrP^C^ distribution or cleavage. Our data provide new insights into the cellular mechanism of prion conversion and suggest that GM1-prion protein interaction at the cell surface could play a significant role in the mechanism predisposing to pathology.

## Introduction

PrP^C^ was first identified as a normal cellular protein almost 30 years ago [1], but its physiological function remains uncertain. The proposed functions of PrP^C^ are related to its localization on the cell surface. Several lines of evidence support the idea that PrP^C^ may play a role in the regulation of ion channels and neuronal excitability; others suggest that PrP^C^ has neuroprotective and pro-survival functions [2]. PrP^C^ is synthesized in the secretory pathway and the mature form is N-glycosylated and anchored to the cell surface by means of a glycosylphosphatidylinositol (GPI)-anchor. GPI-anchored PrP^C^ is present in lipid rafts, microdomains enriched in cholesterol, gangliosides, sphingomyelin and acylated proteins, related to a wide range of biological processes, including intracellular trafficking, transmembrane signalling, lipid and protein sorting, viral uptake and regulated proteolysis [3], [4].

PrP^Sc^ (scrapie prion protein), is the misfolded isoform of PrP^C^ and is the main cause for a group of fatal neurodegenerative disorders known as prion diseases or transmissible spongiform encephalopathies, including Creutzfeldt–Jakob disease, Gerstmann–Sträussler–Scheinker syndrome, fatal familial insomnia and kuru in humans, scrapie in sheep, bovine spongiform encephalopathy in cattle and chronic wasting disease in deer and elk [2]. The key event in the pathogenesis of the prion diseases is the conformational conversion of PrP^C^ into PrP^Sc^, providing the *seed* for biophysical transformation [5].

PrP^C^ has two structurally distinct domains: a flexibly disordered N-terminus which can bind copper ions through the octapeptide repeat region, and a C-terminal globular domain containing three α-helices (HA, HB and HC) and two short antiparallel β-strands [6], [7]. In contrast, PrP^Sc^ is enriched in beta-sheet structures and is characterized by a poor solubility in non-denaturing detergents, propensity for aggregation, and partial resistance to proteinase K digestion [8]. PrP^C^ is absolutely required for the disease processes; PrP^C^ knockout mice fail to develop the disease when inoculated with PrP^Sc^ [9]. Moreover, it is known that endoproteolytic cleavage of PrP^C^ negatively influences prion infection [10], [11]. Two cell surface metalloproteases, A Disintegrin And Metalloprotease (ADAM10 and ADAM17), could be implicated in this process [12], [13] forming a C1 fragment (truncated PrP^C^), that remains membrane-associated, and releasing the soluble, non-toxic and non-amyloidogenic N1 fragment.

Experimental evidence obtained in PrP^Sc^-infected cells suggests that acidic endosomal compartments are relevant sites for prion protein conversion [14], [15], although other subcellular compartments, such as lipid rafts, may also be involved [16]-[18]. *In-vitro* experiments suggest that lipid rafts structure and integrity are essential for the conversion of PrP^C^ into PrP^Sc^, likely by facilitating the contact between misfolded and native protein forms [18]. Consistent with this, rafts disruption (e.g by cholesterol depletion) decreases PrP^Sc^ formation [19], [20]. Whether other rafts components, e.g. gangliosides, may affect such conversion it has not been established. Gangliosides are acidic glycosphingolipids that deeply influence the membrane organization and the function of specific membrane-associated proteins, by lipid-lipid and lipid-protein interactions. It is known that specific gangliosides can interact with selected proteins modulating their function [21], [22] and that administration of exogenous gangliosides displaces GPI-anchored proteins from rafts [23].

At present, however, the role of lipid rafts in PrP^C^ conformational conversion, cleavage and trafficking, is poorly understood.

We investigated whether alterations in lipid rafts ganglioside composition influenced PrP^C^ segregation and processing. Cultured rat cerebellar granule cells (CGCs) were exposed to exogenous gangliosides (GM3, GM1 or GD1a), and prion protein localization in lipid rafts, conformation and proteolytic cleavage were analysed using antibodies against different PrP^C^ epitopes.

The results support the possibility that in neurons at least two PrP^C^ conformations coexist: an α-helical structure, preferentially sequestered within lipid rafts, and a PrP^Sc^-like conformation, resistant to denaturation and predominant in non-raft-membranes and/or intracellular compartments. The increase in the lipid raft ganglioside content does not influence the distribution or the cleavage of PrP^C^. However, the increase of GM1 ganglioside content in lipid rafts remarkably promotes a significant loss of α-helical contents in PrP^C^ inducing a significant structural rearrangement.

## Materials and Methods

### Chemicals

The reagents used (analytical grade) and HPTLC plates (Kieselgel 60) were from Merck, GmbH. Modified Eagle's basal medium, fetal bovine serum, trypsin, 3-[cyclohexylamino]1-propanesulfonic acid (CAPS), 2-[N-morpholino]ethansulfonic acid (MES), ammonium bicarbonate, DAPI and iodoacetamide (IAA) were from (Sigma). Anti-PrP^C^ antibodies (Ab) 8G8 and SAF32 were from Cayman Chemical; anti-PrP^C^ 6H4 from Prionics; anti-ADAM17 and anti-calreticulin antibody (ab2907) from Abcam; anti-PrP^C^ (C20, sc-7693), anti-PrP^C^ (6D11, sc58581), anti-ADAM10 and anti-PKC from Santa Cruz Biotechnology; anti-Thy1 (MAB1406) from Chemicon International; an anti-giantin antiserum was provided by M. Renz (Institute of Immunology and Molecular Genetics, Karlsruhe, Germany). Secondary antibodies for enhanced chemiluminescence (ECL) detection, anti-mouse and/or anti-rabbit-HRP conjugates were from Pierce; PNGasi F from New England Biolabs; Cholerae Toxin B subunit (CTB) conjugate Alexa Fluor 594 and secondary antibodies conjugate with Alexa Fluor 594 and Alexa Fluor 488 were from Invitrogen.

Gangliosides GM3, GM1 and GD1a were prepared from calf brain according to Tettamanti et al. [24]. GM3 ganglioside radiolabelled, [3H]GM3, specific radioactivity 2 Ci-mmol-1, was prepared and purified as described [25]. GM1 and GD1a gangliosides radiolabelled at the level of C-3 of long chain base moiety ([3H]GM1, [3H]GD1a) were prepared and purified as described [26]. Their specific radioactivity was 1.2 Ci/mmol and radioachemical purity was >99%.

### Animals and Cell cultures

Sprague Dawley rats (8 days old) from Charles River (Milan, Italy) were used for this study. Animals were group-housed and received food and water ad libitum. This study was based on protocols (PP04/2006 and PP25/2012) accepted by Italian Ministry of Health (DL 116/92) and by the Veterinarian Responsible to animal care of Medical School (Milano-Bicocca). Following approved protocol, every effort was made to minimize suffering.

Sprague-Dawley rats were anesthetized by isoflurane inhalation (IsoFlurane, Merial), rapidly killed by cervical dislocation, and decapitated using a guillotine. Cerebella was submerged in prewarmed Krebs-Ringer medium (128 mM NaCl, 5 mM KCl, 2.7 mM CaCl2, 1.2 mM MgSO_4_, 1 mM Na_2_HPO_4_, 10 mM glucose, 20 mM Hepes pH 7.35) supplemented with BSA (3 mg/ml) and granule cells in culture (CGCs) were prepared as described [27]. Proliferation of glial cells was prevented by adding cytosine arabinofuranoside (final concentration 10 µM) and checked by microscopic examination. Cell morphology was followed by microscopic examination and cell viability was monitored with fluorescein diacetate and propidium iodide [27]. The experiments were performed with cells cultured for 8 days. The protein content was determined with the micro BCA assay from Sigma Chem. Co. (Milano, Italy).

### Treatment with gangliosides

A given amount of different gangliosides and equivalent tritium-labeled gangliosides (GM3/[^3^H]GM3 or GM1/[^3^H]GM1 or GD1a/[^3^H]GD1a) was dried from a chloroform/methanol (2∶1 by volume) solution and the residue was dissolved in an appropriate volume of Locke's solution to obtain a final 2×10^−6^ M ganglioside concentration with a radioactivity of about 1×10^6^ dpm/mL (ganglioside solution). After removal of the culture medium from each dish, followed by rapid washing and incubation at 37°C for 1 h with Locke's solution to remove FBS, 3 mL of the ganglioside solution was added and incubation was carried out at 37°C for 4 h. In some experiments a lower GM1 ganglioside concentration was used (10^−6^ M at 37°C for 4 h) or a lower temperature (GM1 concentration 2×10^−6^ M at 4°C for 4 h). At the end of incubation, the ganglioside solution was removed, and the cells were washed 3 times with Locke's solution. Cells were then maintained at 37°C for 20 min with 3 mL of FBS-BME; after washing, DRM were prepared following the Triton X-100 method described below. Cell homogenates and each gradient fraction were analysed for radioactive ganglioside content with a liquid scintillation counter.

### Preparation and characterization of Detergent Resistant Microdomains (DRM)

CGCs at 8 DIV, cultured in 100 mm dishes, were washed twice, harvested in Locke's solution (5 mM HEPES pH 7.4, 154 mM NaCl, 5.6 mM Glucose, 5.6 mM KCl, 3.6 mM NaHCO_3_, 2.3 mM CaCl_2_, 1 mM MgCl_2_) and centrifuged. In order to maintain a constant protein/detergent ratio, a cell pellet corresponding to 2.5 mg cellular proteins was incubated in 2 mL of 1% Triton X-100 in 25 mM MES buffer, pH 6.5, containing 150 mM NaCl, 1 mM phenylmethylsulfonyl fuoride (PMSF) and 75 units/mL leupeptin (MBS buffer), for 30 min on ice. The cell lysate was subjected to discontinuous sucrose density gradient centrifugation, as previously described [27], [28]. Briefly, the cell lysate (2 mL) was diluted with an equal volume of 80% (wt/vol) sucrose in MBS lacking Triton X-100 and placed at the bottom of a discontinuous (40–5%, 4 mL each) sucrose concentration gradient in MBS without Triton X-100. After centrifugation at 250,000×*g* for 18 h at 4°C, 1 mL fractions were collected and analysed. The top five fractions of the gradient (mainly fraction 5) contained the DRMs (detergent resistant membranes), as revealed by the enrichment of GM1 ganglioside and cholesterol, as previously published [27].

### Protein analysis

Proteins in the fractions of the sucrose gradient were precipitated with trichloroacetic acid as described [27] and quantified by the micro BCA assay from Sigma Chem. Co. (Milano, Italy). 20 µg of protein was electrophoresed by SDS-PAGE (15% SDS-acrylamide) and electroblotted onto nitrocellulose membranes. Blots were stained with Ponceau S to assess protein loading by densitometry (BIORAD Densitometry 710, program Quantity one) [29]–[30]. Blots were washed with PBS and blocked overnight in PBS-T/milk. After blocking, blots were incubated for 2 h with the primary antibody diluted in PBS-T/milk and then for 2 h with horseradish peroxidase-conjugated anti-rabbit/mouse/IgG (5,000–10,000-fold diluted in PBS-T/milk). Proteins were detected by chemiluminescence (ECL) with the Super Signal detection kit (Pierce, Rockford, IL). Immunoblot bands were analysed and quantified by Kodak Image Station 2000R interfaced with a Kodak Molecular Imaging Software as described [27], [31]. The content of proteins in DRM fractions (control and treated) was normalized to protein loading, as assessed by Ponceau S staining. The enrichment of the protein under investigation in DRM was calculated as previously reported [27], [31].

The data reported for each protein are the mean for 3 immunoblots ± standard deviation (S.D., with different exposure time) obtained from 3 independent sucrose gradients. The significance of the differences was determined by one-way ANOVA and *t*-test.

### Lipid analysis

Lipids were extracted according to Farina et al. [32]. The extracts were analysed by HPTLC. In the case of radioactive ganglioside analysis, the solvent was chloroform/methanol/CaCl_2_ (60/42/11, vol/vol/vol), followed by radioactivity imaging (Beta-Imager 2000 Z Instrument; Biospace, Paris, France). The radioactivity associated with individual gangliosides was determined with the Beta-Vision software (Biospace).

### Protein deglycosylation

50 µg of proteins from all gradient fractions obtained from control and GM1 treated cells, were denatured and incubated with 0.125 U of peptide N-glycosidase F (PNGase F; P0704L, New England Biolabs, MA), at 37°C for 4 h, according to the manufacturer's instructions. The reaction was stopped by adding an equal volume of 2× denaturing buffer (0.125 M Tris, pH 6.8; 4% wt/vol sodium dodecyl sulfate, 10% vol/vol 2-mercaptoethanol, and 20% vol/vol glycerol) [33].

### Immunofluorescence analysis

Cells grown on poly-L-lysine-coated glass coverslips were washed twice with PBS and fixed for 25 min at RT with 4% *p*-formaldehyde and 4% sucrose in 0.12 M sodium phosphate buffer, pH 7.4. Fixed cells were rinsed with PBS, pre-incubated for 20 min in gelatin dilution buffer (GDB: 0.02 M sodium phosphate buffer, pH 7.4, containing 0.45 M NaCl, 0.2% (w/v) gelatin) and 0.3% (v/v) Triton X-100, and incubated with primary antibody in GDB for 2 h at RT. After washing with PBS, coverslips were incubated for 1 h with Alexa Fluor 488- or 594-conjugated secondary anti-mouse or anti-rabbit IgG in GDB, washed with PBS, and incubated for 5 min with 1 µM DAPI in PBS. Immunofluorescence staining was carried out using the following antibodies: monoclonal antibodies directed against PrP^C^ (SAF32, 6H4); polyclonal antibodies against Na^+^/K^+^ ATPase, giantin and calreticulin. Goat anti-mouse Alexa Fluor 488 and 594, goat anti-rabbit Alexa Fluor 488 and 594 (20 µg/ml) and *Cholerae Toxin* B subunit conjugate Alexa Fluor 594.

Coverslips were mounted with a PBS-glycerol solution (1∶9) on glass slides. Images were acquired with Zeiss LSM 710 laser-scanning confocal microscope (Jena, Germany).

### Liposomes preparation

Liposomes were composed of POPC and DDPC mixed or not with GM1, in a 9∶1 molar ratio. Lipids were mixed in chloroform/methanol (2∶1, vol:vol) and dried under a gentle stream of nitrogen followed by a vacuum pump for 3 h to remove traces of organic solvent. The resulting lipid film was rehydrated (at 2 µmol/ml) in acetate buffer (10 mM, pH 5.0), vortexed and then extruded (using a Lipex Biomembranes extrude, Vancouver, Canada) 10 times through a stack of two polycarbonate filter of 100-nm pore size diameter (Millipore) under 20 bar nitrogen pressure [16] Liposomes size, polydispersity index and zeta potential were obtained using a ZetaPlus particle sizer and zeta-potential analyzer (Brookhaven Instruments, Holtsville, New York) at 25°C in acetate buffer by dynamic light scattering with a 652-nm laser beam. The liposome final concentration was 2 mM.

### Circular Dichroism (CD)

Mouse recombinant (rec) PrP^C^ (23–230), prepared as described previously [34], was diluted to a final concentration of 11 µM in 10 mM sodium acetate buffer (pH 5), in the presence or absence of 1 mM liposomes of different composition (POPC, POPC-GM1, DPPC and DPPC-GM1). CD-spectra were collected for each sample with a Jasco J-815 spectropolarimeter (Jasco, Easton, USA), using a 260–190 nm wavelength range, in a 0.1 cm path length quartz cell. All spectra were acquired with a bandwidth of 1.0 nm and a resolution of 0.1 nm. Temperature was maintained at 22°C with a Peltier heating system (Jasco). An average of five scans was obtained for each sample, with a sensitivity of 100 mdeg, a response of 4 sec and a scan speed of 50 nm/min. CD spectra, subtracted for buffer signal, were expressed as mean molar ellipticity (Φ).

## Results

### PrP^C^ distribution in CGCs

**Figure 1:**
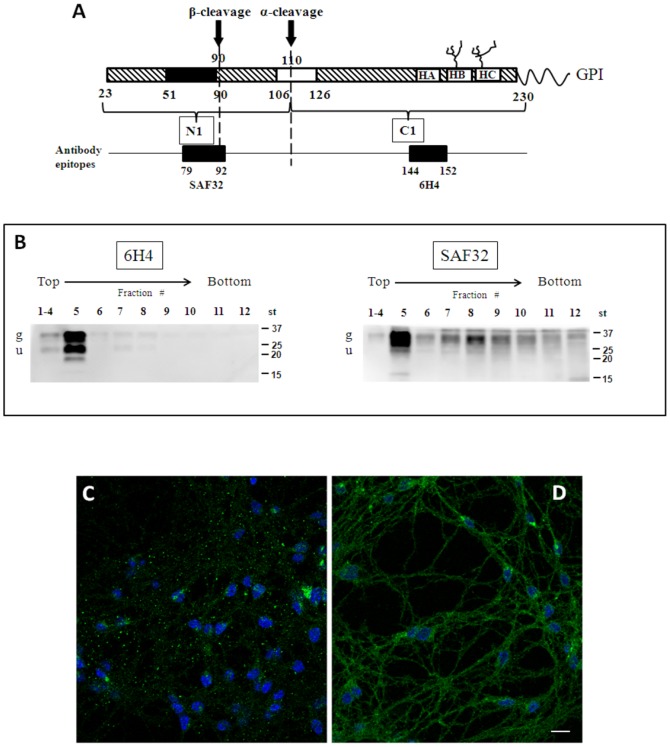
Characterization of PrP^C^ distribution in CGCs. Panel A- Schematic diagram of the proteolysis of PrP^C^ and the epitope recognized by the antibodies used in this study. The native full-length PrP^C^ is shown with its C-terminal GPI-anchor, the two N-linked glycosylation sites, the three helical regions (HA, HB and HC), the octapeptide repeat region (black), and the “toxic” 106–126 domain (white). The epitopes for antibody SAF32 (residues 79–92, in the unstructured octapetidic stretch) and 6H4 (residues 114–152, localized in HA) are indicated. The two cleavage sites generating N1/C1 (α-cleavage) and N2/C2 (β-cleavage) are shown by arrows. C1 is recognized only by 6H4. Panel B Characterization of PrP^C^ localization in gradient fractions prepared from control CGCs. Cells were incubated with 1% Triton X-100-containing buffer for 30 min on ice. The suspension was subjected to discontinuous sucrose density gradient centrifugation. One-milliliter fractions were withdrawn from the gradient, submitted to 15% SDS-PAGE, transferred to nitrocellulose membranes, and immunoblotted with 6H4 or SAF32 antibodies against PrP^C^ (20 µg proteins/lane), followed by ECL detection. Representative blots from three independent experiments are shown. g: glycosylated PrP^C^; u: unglycosylated PrP^C^. Panel C and D: immunofluorescence images showing PrP^C^ distribution visualized by 6H4Ab (C) and SAF32Ab (D) in 8 DIV CGCs. Note the clusterized pattern visualized by 6H4Ab with respect to the diffuse staining of SAF32Ab. DAPI staining (blu) evidences nuclei. Scale bar: 10 µm.

To monitor the enrichment of PrP^C^ in lipid rafts, we fractionated cold-detergent lysates of CGCs by sucrose gradient and analyzed the fractions by immunoblotting with either 6H4Ab, that recognizes an epitope between residues 144–152 (within α-helix HA), or SAF32Ab that recognizes residues 79–92 in the octapetide repeat region (Fig. 1A).

Immunoblotting analysis of gradient fractions with 6H4Ab indicated that PrP^C^ was strongly enriched in DRMs (Fig. 1B). Two main bands were detected: one at 35 kDa, corresponding to the full-length (f.l.) glycosylated PrP^C^ and one at 25 kDa corresponding to unglycosylated PrP^C^ [35]. In contrast, SAF32Ab preferentially revealed the PrP^C^ glycosylated isoform at 35 kDa, that was present in all gradient fractions, besides being enriched in DRMs (Fig. 1B).

**Figure 2:**
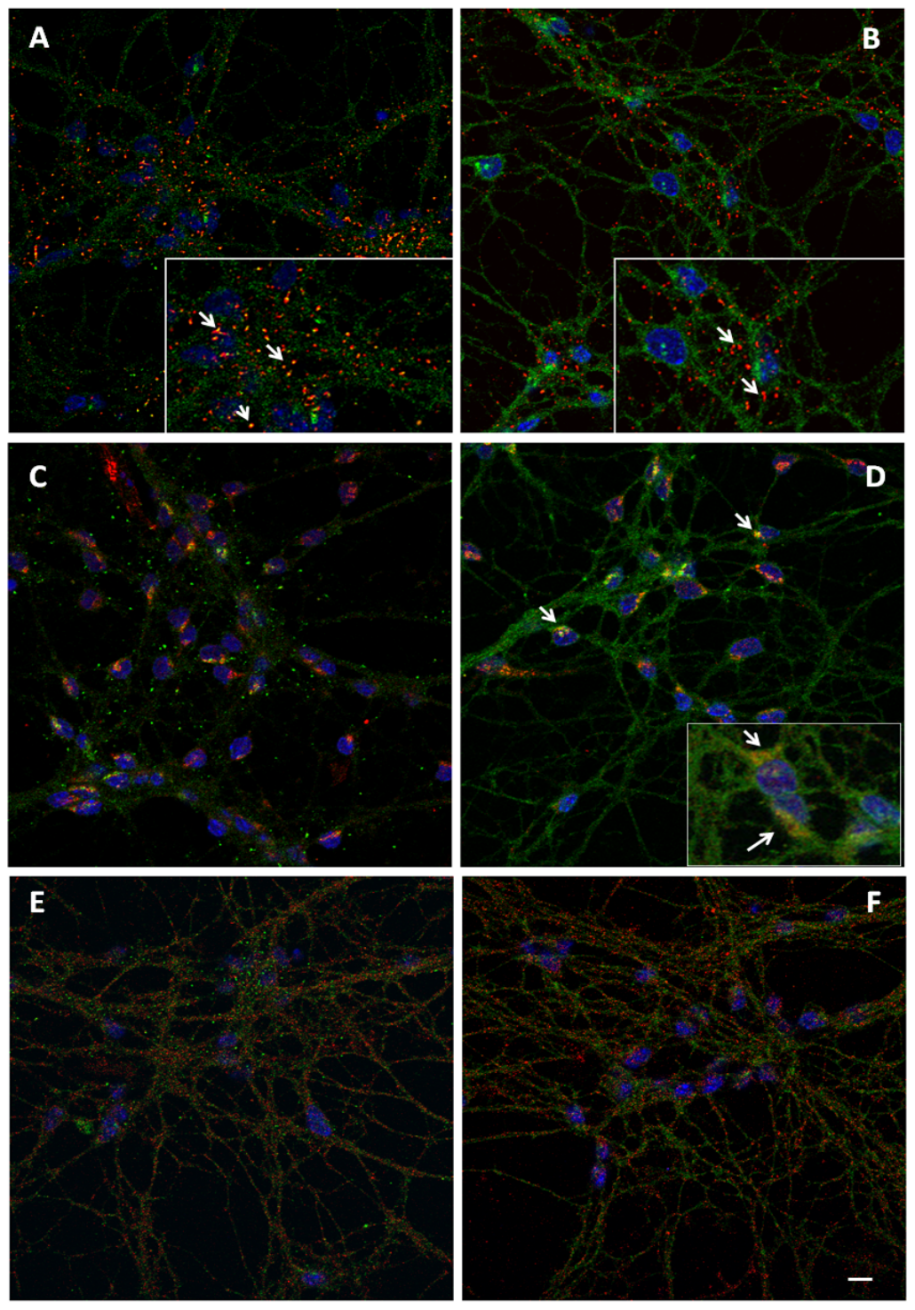
Immunofluorescence analysis of PrP^C^ distribution in CGCs. Panel A: CGCs were immunolabelled with PrP^C^ 6H4Ab (green) and the Alexa Fluor 594 cholerae toxin B (red) to visualize lipid rafts. Panel B: CGCs were doubly immunolabelled with PrP^C^ SAF32 (green) and *cholerae toxin* B subunit (CTB). Insets show the different colocalization of the Abs with lipid rafts, indicating that 6H4Ab preferentially recognizes PrP^C^ resident in lipid rafts, while SAF32Ab show PrP^C^ that is widespread throughout the membrane. Arrows mark the position of CTB. Panel C and D: double immunofluorescence of PrP^C^ antibodies with Giantin (red) denoting a major presence of SAF32Ab-positive PrP^C^ in the Golgi apparatus with respect to 6H4Ab. Arrows mark the colocalization. Panels E and F: double immunofluorescence of PrP^C^ antibodies (green) showing a lack of colocalization with Na^+^-K^+^/ATPase, a non-lipid raft plasmamembrane marker (red). Scale bar: 10 µm.; insets: 20 µm.

To test whether the 6H4 and SAF32 antibodies detected PrP^C^ molecules in different cellular compartments, we analyzed CGCs by immunofluorescence confocal microscopy. Immunostaining with 6H4Ab showed clusters of PrP^C^ on the cell bodies and neuritis (Fig. 1C), while SAF32Ab showed a more diffuse distribution (Fig. 1D). Labeling with *Cholerae Toxin B* subunit (CTB), a widely used lipid rafts marker [36], showed strong colocalization with the 6H4Ab-positive clusters (Fig. 2A), whereas CTB colocalized with SAF32Ab to a much lesser extent (Fig. 2B). Double immunolabeling with anti-PrP^C^ and anti-giantin antibodies [37] showed some SAF32Ab-reactive PrP^C^ also in the Golgi (Fig. 2C and 2D). Prion protein did not colocalize with Na^+^-K^+^/ATP-ase, a non-lipid rafts plasmamembrane marker [38], tested either with SAF32Ab or 6H4Ab (Fig. 2E and Fig. 2F). Finally, both Abs did not colocalize with calreticulin, a marker of the endoplasmic reticulum (Fig. S1).

Taken together, the biochemical and immunofluorescence analyses suggest that 6H4Ab preferentially recognizes a form of PrP^C^ enriched in lipid rafts, while SAF32Ab detects PrP^C^ molecules in both lipid rafts and other membrane compartments.

### Effect of ganglioside treatment on the localization of PrP^C^ in gradient fractions

To test the effect of gangliosides on PrP^C^ distribution between raft and non-raft membrane regions, CGCs were incubated with different radiolabelled gangliosides ([^3^H]GM3 or [^3^H]GM1 or [^3^H]GD1a) at a final concentration of 2×10^−6^ M, for 4 h at 37°C followed by 30 min washing with FBS (Standard treatment, St). After this treatment, the amount of gangliosides associated with the cells was constituted mainly by serum stable form of plasma membrane-associated gangliosides [25].

**Figure 3:**
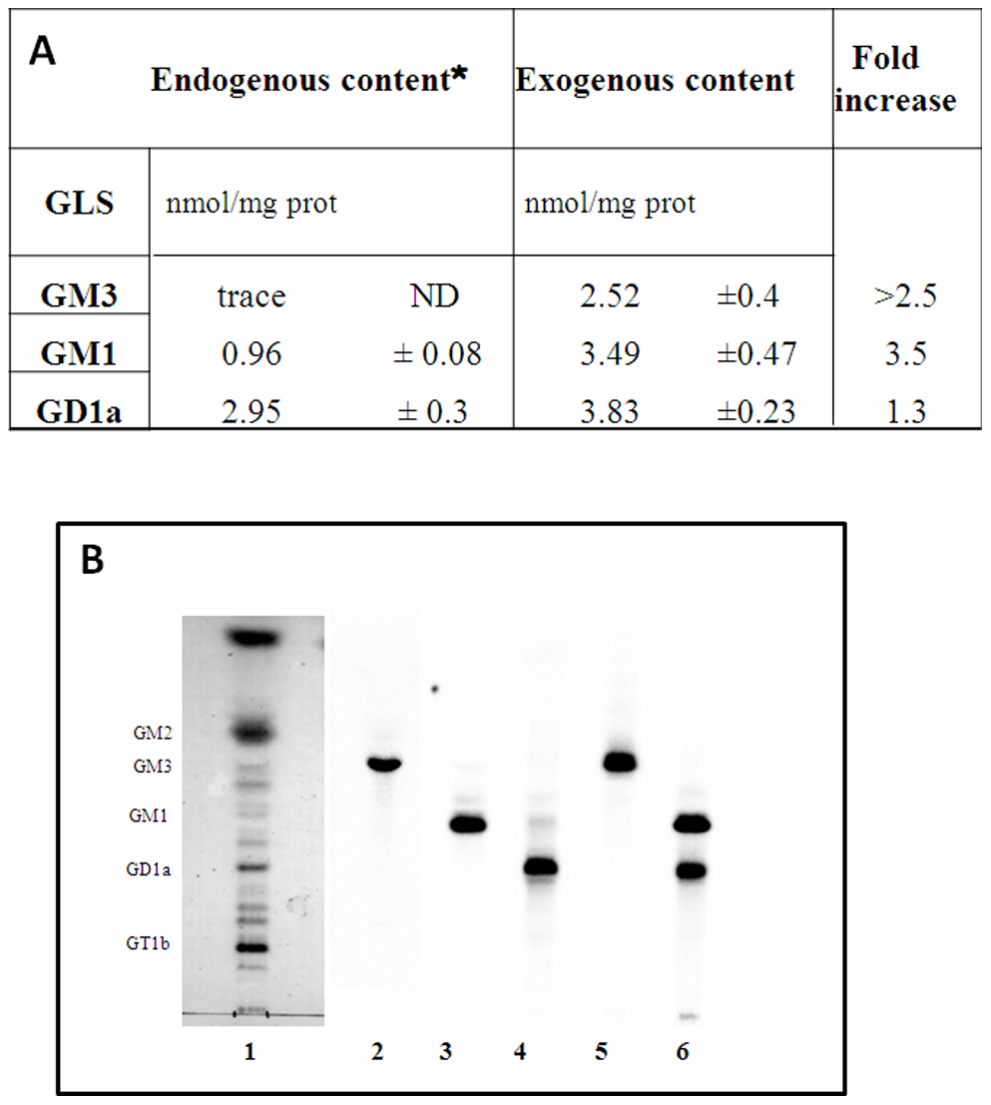
Cellular gangliosides treatment. CGCs were incubated with different gangliosides (GM3, GM1 or GD1a) and correspondent radiolabelled gangliosides ([^3^H]GM3, [^3^H]GM1 or [^3^H]GD1a), at a final concentration of 2×10^−6^ M at 37°C for 4 h. At the end of incubation, the ganglioside solution was removed and cells were washed 3 times with Locke's solution and maintained at 37°C for 20 min in 3 mL of FBS-BME. The lipids extract, from cell homogenates, were analysed to determine the ganglioside incorporation (panel A) and metabolism by HPTLC following radioactivity imaging (panel B). Lane 1: granule cell ganglioside pattern; lane 2: granule cells ganglioside extracted after incubation with GM3/[^3^H]GM3 2×10^−6^ M at 37°C for 4 h; lane 3: granule cells ganglioside extracted after incubation with GM1/[^3^H]GM1 2×10^−6^ M at 37°C for 4 h; lane 4: granule cells ganglioside extracted after incubation with GD1a/[^3^H]GD1a 2×10^−6^ M at 37°C for 4 h; lane 5: [^3^H]GM3 standard; lane 6. [^3^H]GM1 and [^3^H]GD1a standards. * Gangliosides endogenous content as reported by Palestini et al., 1991. [40]

The total ganglioside incorporation, compared to the endogenous content [27], [32] is reported in Figure 3A. Although the total incorporation of the different gangliosides was very similar, the plasma membrane ganglioside concentration increased differently (GM3>GM1>GD1a). Figure 3B shows the endogenous ganglioside pattern (lane 1) and radioactive gangliosides extracted from treated-CGCs homogenates (lane 2–4), showing that after ganglioside treatment the amount of [^3^H]-metabolites was less than 5%.

**Figure 4:**
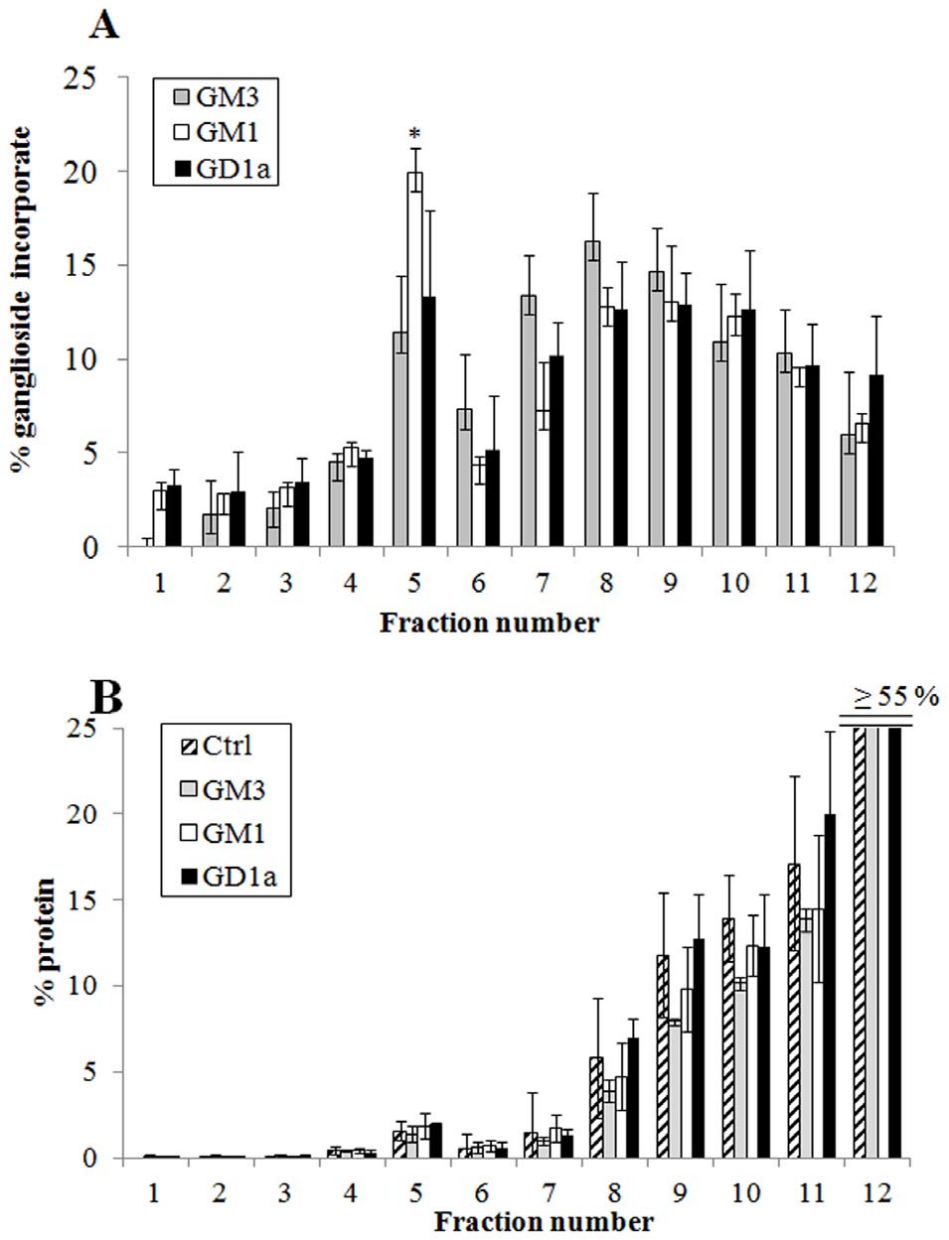
Distribution of gangliosides radioactivity and proteins in the different fractions of the sucrose gradient. CGCs, after incubation with different gangliosides (GM3, GM1 or GD1a) and correspondent radiolabelled gangliosides ([^3^H]GM3, [^3^H]GM1 or [^3^H]GD1a), at a final concentration of 2×10^−6^ M at 37°C for 4 h, were treated with 1% Triton X-100-containing buffer for 30 min on ice. The cellular lysate was submitted to discontinuous sucrose density gradient centrifugation. One-milliliter fractions were withdrawn from the gradient and submitted to [^3^H]GM3, [^3^H]GM1 or [^3^H]GD1a radioactivity determination (panel A) and evaluation of proteins distribution (panel B). Data are means ± SD from at least three independent experiments performed in triplicate.

Figure 4A reports the percentage incorporation of GM3, GM1 and GD1a in the different subcellular fractions, obtained from CGCs incubated with the three gangliosides. GM1 was found mainly in fraction 5, (20% of the total) corresponding to detergent resistant membranes (DRMs), being lower in fractions 6–12. GM3 or GD1a were more homogeneously distributed. The protein distribution in the different gradient fractions of ganglioside treated-cells did not change significantly compared to control cells (Figure 4B), with 1.6% of proteins being in DRMs in both cases.

**Figure 5:**
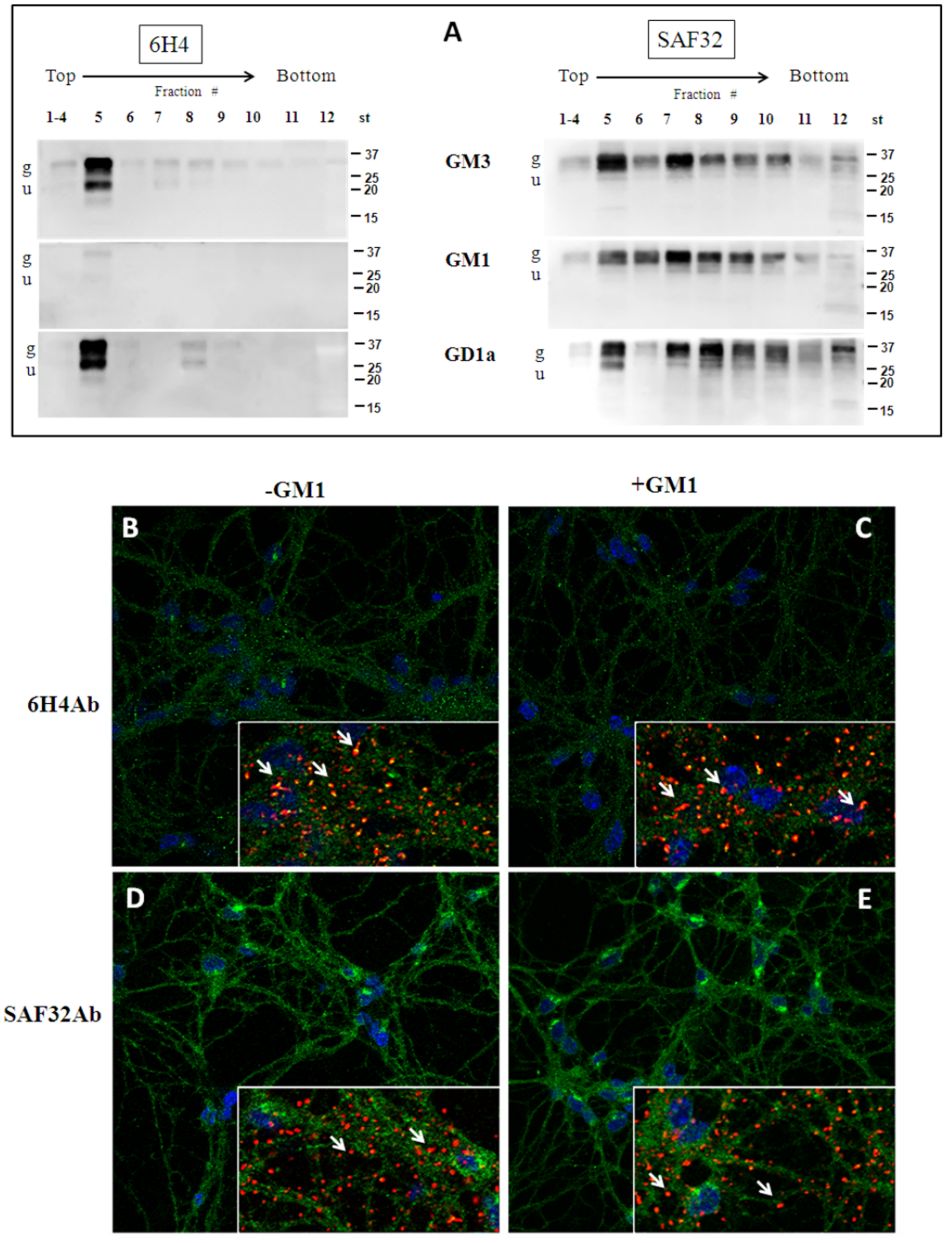
Characterization of PrP^C^ in gradient fractions obtained from treated CGCs. Panel A Cells, after incubation with different gangliosides (GM3, GM1 or GD1a) and correspondent radiolabelled gangliosides ([^3^H]GM3, [^3^H]GM1 or [^3^H]GD1a), at a final concentration of 2×10^−6^ M at 37°C for 4 h (Standard treatment, St), were treated with 1% Triton X-100-containing buffer for 30 min on ice. The cellular lysate was submitted to discontinuous sucrose density gradient centrifugation. One-milliliter fractions were withdrawn from the gradient, submitted to 15% SDS-PAGE (20 µg protein/lane), transferred to nitrocellulose membranes and immunoblotted with 6H4 or SAF32 antibodies against PrP^C^ followed by ECL detection. Representative blots from three independent experiments are shown. g: glycosylated PrP^C^; u: unglycosylated PrP^C^. GM3 = GM3-treated CGCs; GM1 = GM1-treated CGCs; GD1a = GD1a-treated CGCs. Panel B-E: immunofluorescence analysis of PrP^C^ in CGCs with 6H4Ab (B and C) and SAF32Ab (D and E) in the presence (C and E) or in the absence (B and D) of GM1. Note that after ganglioside treatment, PrP^C^ recognized by 6H4Ab appears generally less clusterized being more widespread and less concentrated around cell bodies and proximal dendrites (C, green), while PrP^C^ distribution detected by SAF32Ab does not differ from that of control cells. Insets show double staining of PrP^C^ and CTB. Arrows mark the position of CTB. Scale bar: 10 µm; insets: 20 µm.

Next, we investigated the distribution of PrP^C^ in gradient fractions from CGCs treated with the different gangliosides (Fig. 5A). Surprisingly, immunoblotting with 6H4Ab failed to detect PrP^C^ in the gradient fractions of GM1-treated-cells, with a very faint signal only in fraction 5 (compare to Fig. 1B showing the same analysis of untreated cells). In contrast, GM3 and GD1a did not have any effect on the reactivity of this antibody. When SAF32Ab was used to immunodetect PrP^C^, the protein distribution in GM3-, GM1- and GD1a-treated cells did not differ from that of untreated cells (Fig. 5A and 1B).

Confocal microscopy analysis with CTB and SAF32Ab on GM1-treated CGCs showed that the PrP^C^ distribution and colocalization with GM1 did not change with respect to control cells (Fig. 5D and 5E). On the other hand, 6H4Ab showed that PrP^C^ was less clusterized around cell bodies and proximal dendrites (Fig. 5B and 5C, green) and colocalized less with GM1 domains with respect to controls (Fig. 5B and 5C, inset).

Incubation of CGCs with GM3 or GD1a did not alter the colocalization of CTB and PrP^C^, as detected with either antibody (Fig. S2).

### Effect of ganglioside treatment on the localization of PKC, ADAM10, ADAM17 and Thy1 in gradient fractions

**Figure 6:**
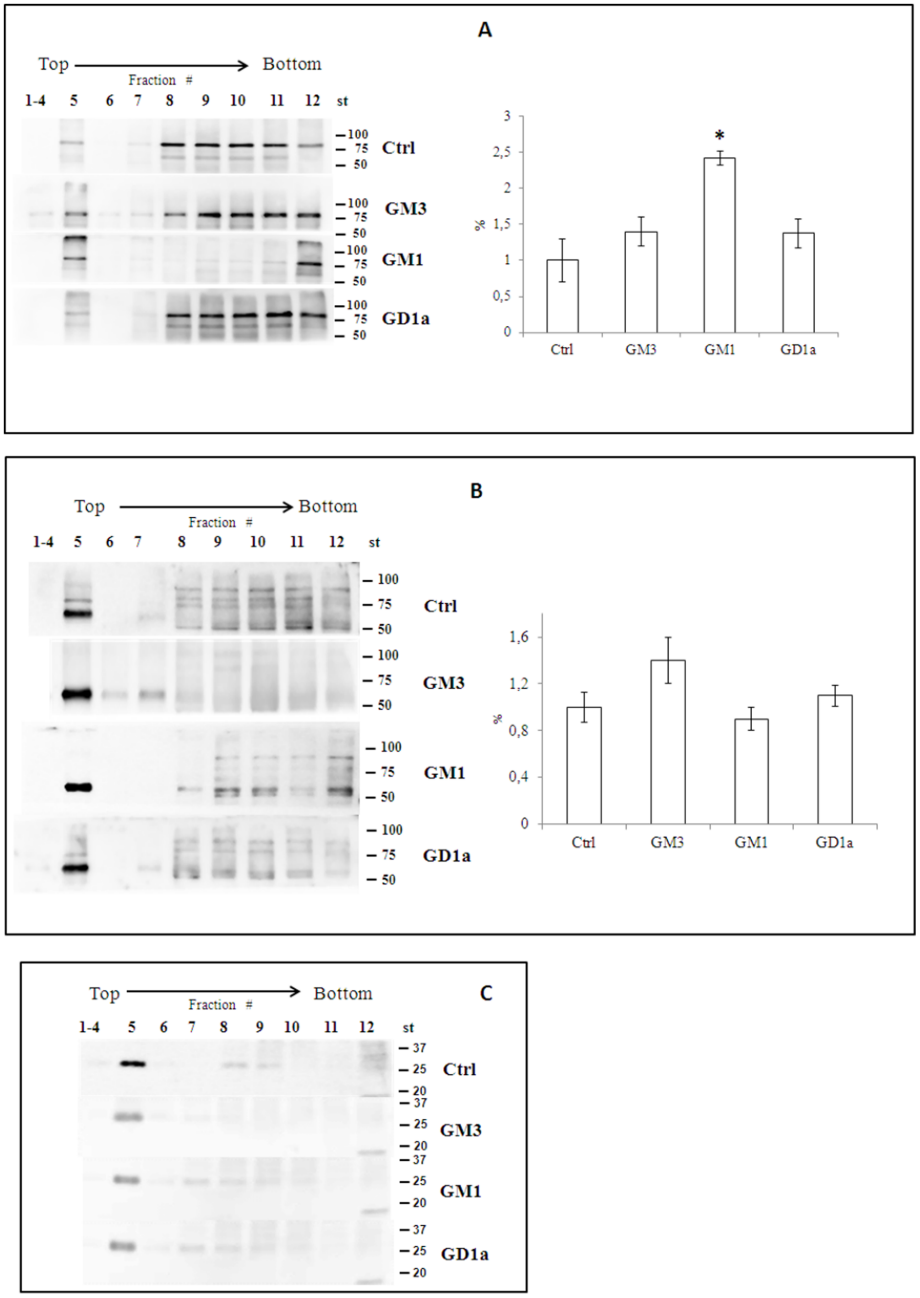
Effect of gangliosides treatment on the localization of PKC, ADAM10 and Thy1 in gradient fractions from gangliosides treated-CGCs. Cells, after incubation with different gangliosides (GM3, GM1 or GD1a) and correspondent radiolabelled gangliosides ([^3^H]GM3, [^3^H]GM1 or [^3^H]GD1a), at a final concentration of 2×10^−6^ M at 37°C for 4 h, were treated with 1% Triton X-100-containing buffer for 30 min on ice. The cellular lysate was subjected to discontinuous sucrose density gradient centrifugation. One-milliliter fractions were analyzed by immunoblotting with anti-PKC (panel A), anti-ADAM10 (panel B) and anti-Thy1 (panel C) antibodies. Immunoblot bands were analyzed and quantified by Kodak Image Station 2000R interfaced with a Kodak Molecular Imaging Software. The enrichment of the proteins in DRM was calculated as previously reported [27]. The data reported for each protein are the mean of 3 immunoblots ± S.D. obtained from 3 independent sucrose gradients. Ctrl =  CGCs control; GM3 = CGCs treated with GM3; GM1 =  CGCs treated with GM1; GD1a =  CGCs treated with GD1a. Ctrl *vs* GM1 *p<0.01 (one way ANOVA).

We investigated whether treatment with exogenous gangliosides affected the distribution of PKC, ADAM10 and ADAM17 within raft and non-raft regions (Fig. 6). A significant enrichment of PKC in DRMs was observed after GM1 treatment (Fig. 6A). ADAM10 was enriched in DRMs of untreated CGCs, and its localization did not change after GM3, GM1 or GD1a treatment (Fig. 6B). ADAM17 was detectable only outside DRMs and its distribution did not change after treatment (Fig. S3).

The distribution of Thy1, a neuronal GPI-anchored protein [32] that is not substrate of ADAMs or PKC, was also analysed. This protein was particularly enriched in DRMs and its distribution did not change after ganglioside treatment (Fig. 6C).

### PNGase F treatment

**Figure 7:**
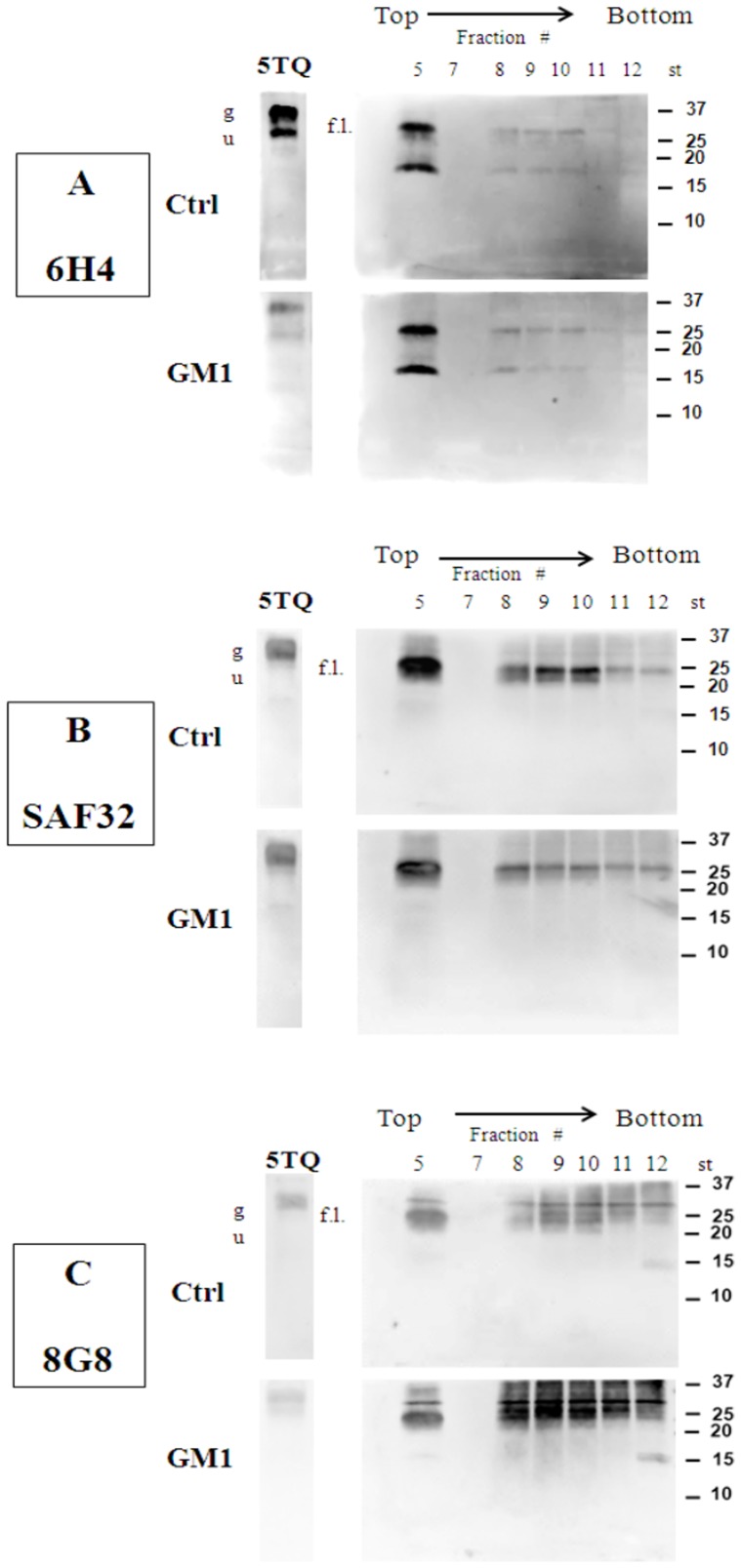
Influence of GM1 cells treatment in PrP^C^ processing. CGCs, after incubation with GM1/[^3^H]GM1 at a final concentration of 2×10^−6^ M at 37°C for 4 h, were treated with 1% Triton X-100-containing buffer for 30 min on ice. The cellular lysate was submitted to discontinuous sucrose density gradient centrifugation. 50 µg of proteins from gradient fractions were subjected to protein deglycosylation by PNGase F treatment and immunoblotted with 6H4Ab (panel A), SAF32Ab (panel B) and 8G8Ab (panel C). Bands were analyzed and quantified by Kodak Image Station 2000R interfaced with a Kodak Molecular Imaging Software. Representative blots from three independent experiments are shown. 5TQ =  fraction 5 not subjected to PNGase F treatment; f.l.  = full length-PrP^C^; u  =  unglycosylated PrP^C^; g =  glycosylated PrP^C^.

Additional experiments were done to assess whether GM1 modified the endoproteolytic cleavage of PrP^C^ by ADAMs. The normal constitutive cleavage (α-cleavage) of PrP^C^ leads to the formation of a soluble N-terminal fragment (N1) and C-terminal fragment (C1) that remains attached to the membrane, while β-cleavage generates N2 (soluble) and C2 (membrane attached) fragments (Fig. 1A). Full-length (f.l.) and truncated forms of PrP^C^ were separated by EF and analyzed by WB after protein deglycosylation with PNGaseF [39]. 6H4Ab detected two bands of similar intensity in untreated CGCs, one with an apparent molecular mass of about 27 kDa, corresponding to f.l. PrP^C^, and another at about 18 kDa, likely corresponding to the C1 fragment. In GM1-treated-CGCs, the same bands were visible after deglycosylation (Fig. 7A), in clear contrast to samples not subjected to deglycosylation, where PrP^C^ was poorly detectable (compare with Fig. 5A). In contrast, SAF32Ab detected only one band at 27 kDa, corresponding to the deglycosylated full-length PrP^C^ (Fig. 7B). These results indicate that GM1 treatment does not affect the cleavage of PrP^C^ by ADAMs when compared to control CGCs.

To characterize the PrP^C^ 18 kDa band better, we immunoblotted the same samples with the 8G8 antibody (8G8Ab). This antibody is against region 95–110 of PrP^C^, and detects only the C2 fragment derived from β-cleavage [39] but not the C1 fragment. The 18 kDa band did not react with the 8G8Ab (Fig. 7C), confirming that it corresponded to the C1 fragment. Thus, approximately 50% of PrP^C^ in CGC DRMs was α-cleaved.

### Temperature and GM1 dose dependence of PrP^C^ distribution in GM1-treated CGCs

**Figure 8:**
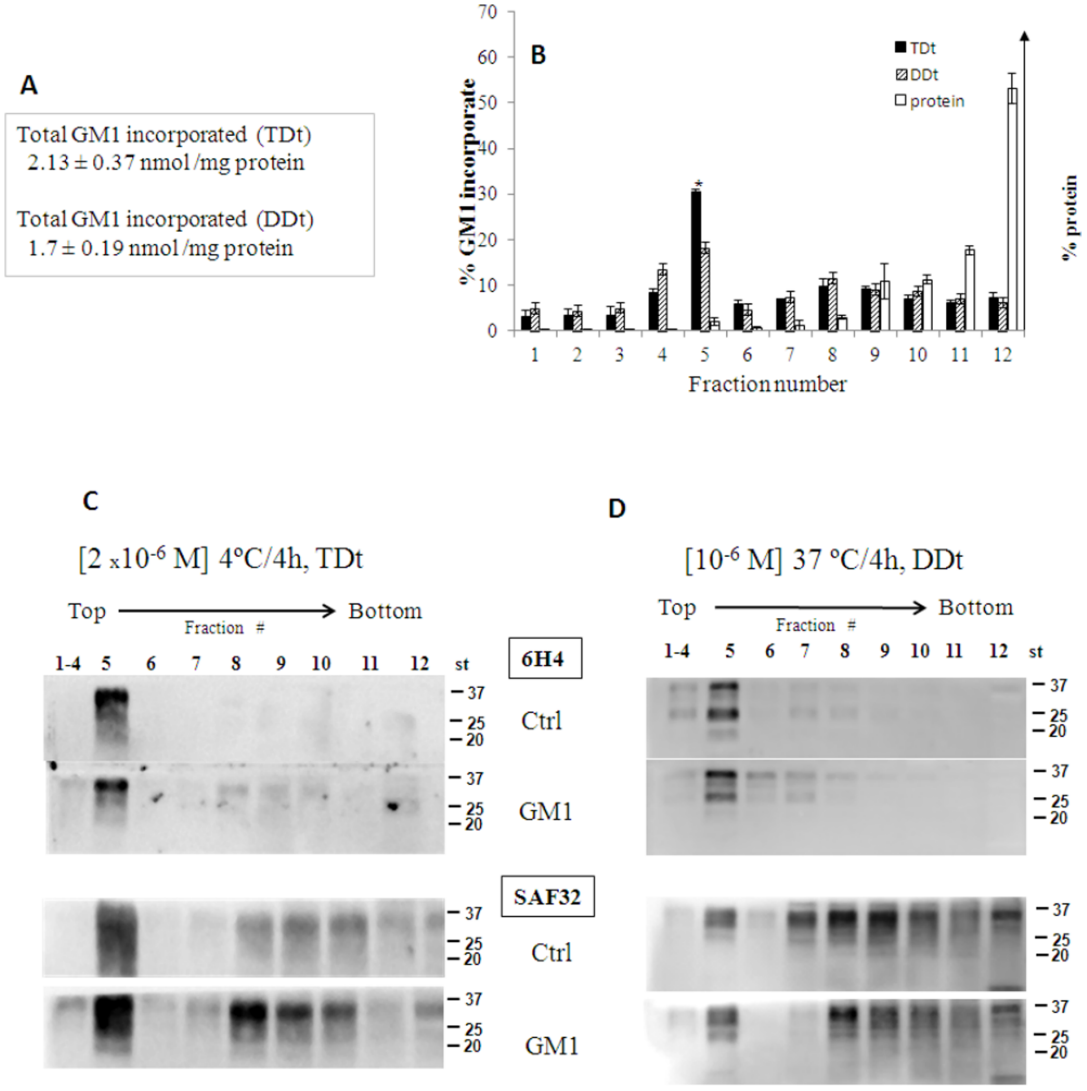
Temperature and GM1 dose dependence of PrP^C^ distribution in GM1-treated CGCs. Cells after incubation with GM1/[^3^H]GM1 2×10^−6^ M at 4°C for 4 h or 1×10^−6^ M at 37°C for 4 h, were treated with 1% Triton X-100-containing buffer for 30 min on ice. A small amount of cells homogenates were analyzed to determine the gangliosides incorporation (panel A) and the residual was submitted to discontinuous sucrose density gradient centrifugation. One-milliliter fractions were withdrawn from the gradient and submitted to proteins and [^3^H]GM1 radioactivity determination (panel B). 20 µg of proteins from different fractions were submitted to 15% SDS-PAGE, transferred to nitrocellulose membranes and immunoblotted with 6H4Ab or SAF32Ab followed by ECL detection (panel C and D). Immunoblot bands were analyzed and quantified by Kodak Image Station 2000R interfaced with a Kodak Molecular Imaging Software. Representative blots from three independent experiments are shown. Ctrl  = control CGCs; GM1  =  GM1-treated CGCs.

CGCs were treated with a lower dose of GM1 (10^−6^M at 37°C, Dose-Dependent treatment, DDt) or with the same dose used in previous experiments (2×10^−6^ M) at 4°C (Temperature-Dependent treatment, TDt) and GM1 incorporation and effect on PrP^C^ distribution in DRMs were compared to the standard treatment, St (2×10^−6^ M at 37°C). The total GM1 incorporation (3.5 nmol/mg protein in St) was reduced to about 2.1 and 1.7 nmol/mg protein in TDt and DDt, respectively (Fig. 8). After TDt, the GM1 incorporation in DRMs was higher as compared to DDt (30% and 18%, respectively) and in both cases the ganglioside incorporation in gradient fractions 6–12 was very low (Fig. 8A). In either treatment [^3^H]-metabolites were not detected (data not shown).

Next, we compared the PrP^C^ distribution in the gradient fractions after TDt and DDt by means of 6H4Ab and SAF32Ab (Fig. 8C and 8D). 6H4Ab-reactive PrP^C^ bands were clearly visible and enriched in DRMs after both DDt and TDt, while PrP^C^ was poorly detectable after St (see Fig. 5A). In the case of SAF32Ab, PrP^C^ was detectable in all gradient fractions, although mostly enriched in fraction 5, similar to that observed after St (compare Figures 1A and 5A). These results indicate that the effects of GM1 become overt only under conditions of higher ganglioside incorporation in DRMs (≥0.7 nmol/mg protein), at physiological temperature.

Confocal analyses of control CGCs and GM1-treated cells showed a very similar PrP^C^ distribution after either TDt or DDt (Fig. S4), in contrast to GM1-treated CGCs under after St (Fig. 5A and 5B).

### Binding to GM1-containing liposomes promotes PrP^C^ misfolding

**Figure 9:**
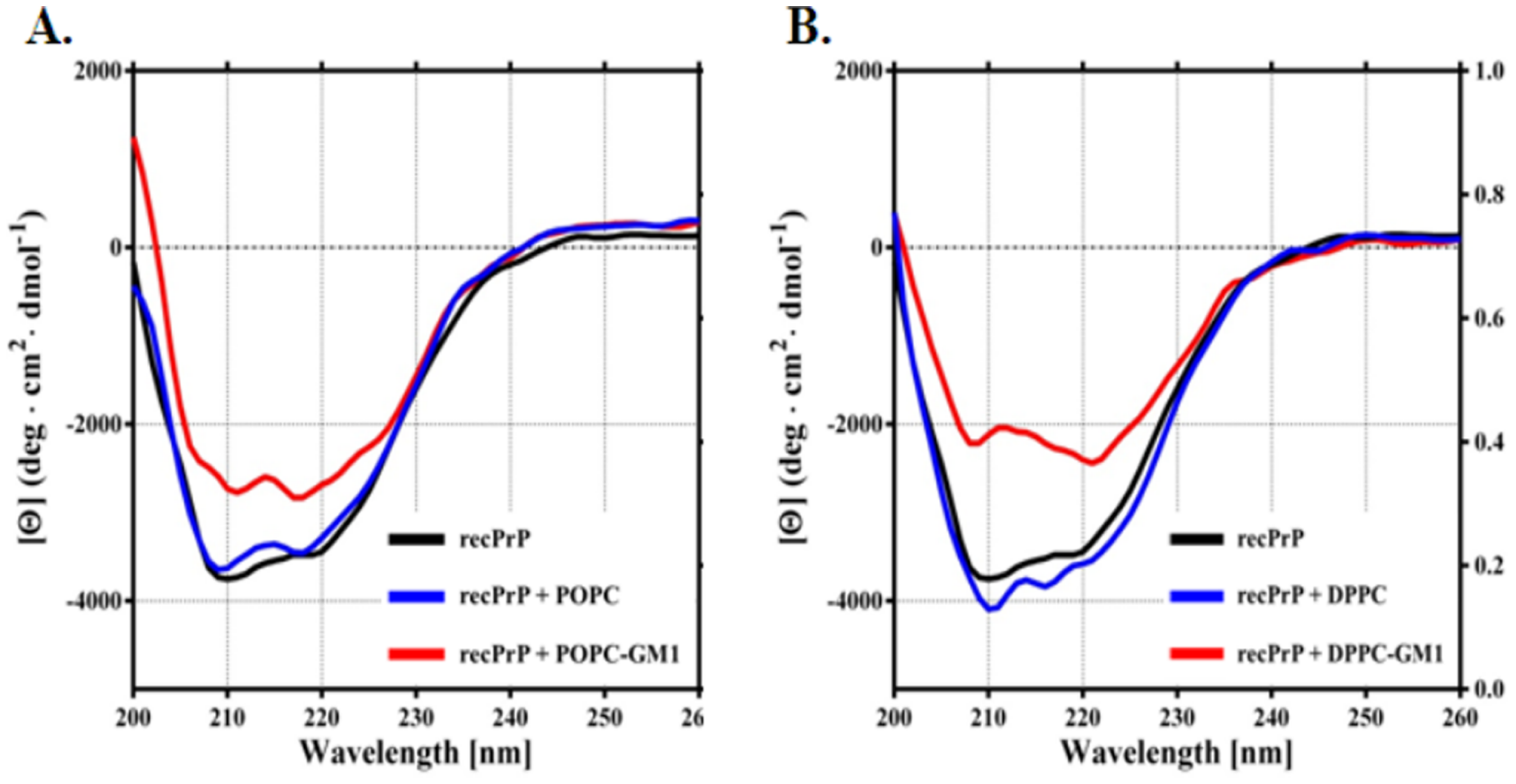
GM1-containing liposomes alter PrP^C^ structure. Panel A- Circular dichroism spectra of recPrP (23–231) alone (black line) or mixed with POPC liposomes (blue line) or GM1-containing POPC liposomes (red line). Panel B- Results obtained replacing POPC with DPPC liposomes.

To directly test whether interaction with GM1 altered the native folding of PrP^C^, we incubated recPrP (23–230) with POPC or DPPC liposomes containing the GM1 ganglioside. The secondary structure of recPrP was then analysed by CD. The CD spectrum of recPrP alone showed typical minima at 208 and 220 nm, indicating the presence of substantial amount of α-helical structures (Figure 9, black line). The CD profile was slightly altered when recPrP was co-incubated with POPC (Figure 9A, blue line) or DPPC (Figure 9B, blue line) liposomes, although no differences in the total content of α-helical structures were detected. These results indicate that a weak interaction may occur between recPrP and POPC or DPPC liposomes, without altering the secondary structure of the protein. In contrast, the spectra of recPrP mixed with GM1-containing POPC liposomes (POPC-GM1) showed a marked alteration of the CD profile, with significant loss of α-helical contents (Figure 9A, red line). An even greater effect was observed when the GM1 ganglioside was incorporated into DPPC liposomes (Figure 9B, red line). These results indicate that the presence of GM1 ganglioside increases the affinity of recPrP for POPC or DPPC liposomes, and induces a significant structural rearrangement in the protein.

## Discussion

Cerebellar granule cells (CGCs) are a well characterized *in-vitro* neuronal model to study the role of gangliosides and prion protein in membrane functionality [40], [41]. Differentiated granule cells at 8 days in culture display a complex ganglioside pattern reaching the standard content of 10.8 nmol/mg protein (as lipid-bound sialic acid) [40]. Lipid rafts of CGCs are well characterized also in terms of protein components, in particular prion protein. In previous studies, we have shown that PrP^C^ is localized in a specific subtype domain in CGC-derived DRMs (Prion Domain, *PrD*), showing typical sphingolipid composition and containing proteins involved in synaptic plasticity, cell adhesion, cytoskeleton regulation and signaling [27], [32].

Several reports suggest that conversion of PrP^C^ into PrP^Sc^ takes place in these lipid microdomains where PrP^Sc^ acts as molecular template, physically interacting with PrP^C^ and converting the latter to PrP^Sc^ [42]. This process can be modulated by modifications of the lipid environment [43]–[45]. In particular, it was shown that modification of lipid rafts composition affects the PrP^C^ localization [46]. Other essential requirements for the conversion to PrP^Sc^ are the integrity and accessibility of toxic domain in PrP^C^ [10], [13].Even if the absence of the toxic domain of PrP^C^ might not completely prevent transmissible spongiform encephalopathy, it may slow down the infection. Therefore, PrP^C^ truncation could be considered protective against prion propagation [47].

On the basis of these considerations, the purpose of this study was to investigate the effect of increasing cell ganglioside content on the membrane segregation and processing of PrP^C^ in CGCs.

It is known that under slightly destabilizing conditions, PrP^C^ can form a β-structured state similar to PrP^Sc^ (PrP^Sc^-like conformation)[48]–[51]. Hornemann and Glockshuber [50] proposed that the β-state is a monomeric transitional folding that lays between the native and the PrP^Sc^ state. Khan et al. and Zou and Cashaman [52], [53] demonstrated that slight changes in physicochemical parameters of the solution (pH, ionic strength and denaturant concentration) can greatly influence the population of β-structured monomers. The data from the literature [14], [15] indicate that, *in-vitro*, the PrP^Sc^-like conformation is thermodynamically stable both at acidic pH present in intracellular compartments and at the neutral pH of the cell surface. Moreover, the PrP^Sc^-like conformation is resistant to denaturation and proteinase K digestion.

For our investigation we used a set of antibodies that recognize different epitopes or different folding states of prion protein. Specifically, 6H4Ab has been repeatedly demonstrated to recognize an epitope between residues 144–152 in α-helix HA, which is accessible in native PrP^C^ but not in PrP^Sc^-like molecules. Korth et al. showed that 6H4Ab specifically recognizes native PrP^C^ but not native PrP^Sc^ [54]. Moreover, Cordes et al. found that introducing a denaturing and/or disaggregating step increases 6H4Ab binding to PK-resistant PrP [55]. Furthermore immunoprecipitation of normal and CJD brain samples showed that 6H4Ab recognizes native PrP^C^ but not native PrP^Sc^, suggesting that residues 144–152 that constitute the 6H4 epitope are buried in PrP^Sc^-like conformation.

In contrast, the mouse anti-PrP monoclonal antibody SAF32 binds to prion protein independently of its conformation since its binding epitope is located at the N-terminal octarepeat region [56].

In line with data suggesting that PrP^C^ might assume different conformations [14], [48], [57], our results support the idea that in CGCs, distinguishable α-helical and PrP^Sc^-like conformations coexist.

Using 6H4Ab we found that the native α-helical PrP^C^ is enriched in DRMs, whereas SAF32Ab detected PrP^C^ in all sucrose gradient fractions. Since SAF32Ab recognizes an epitope (amino acids 23–121) localized in the flexible “unstructured N-terminal tail”, these results suggest that all gradient fractions contain both the native and the PrP^Sc^-like (resistant to denaturation) conformers of PrP^C^, while DRMs are particularly enriched in α-helical PrP^C^.

Indeed immunofluorescence with 6H4Ab showed a clustered immunopositive signal that was enriched in lipid rafts. Instead, SAF32Ab detected PrP^C^ either in the Golgi and in non-raft membrane domains. Similar results were obtained with antibodies C20 and 6D11 whose epitopes are similar to those of 6H4Ab and SAF32Ab, reinforcing our conclusions (Fig. S5).

All these data advocate that α-helical PrP^C^ is enriched in DRMs, while the other membrane compartments contain also molecules with PrP^Sc^-like conformations.

Evidence indicates that the composition of lipid rafts influences the conversion of PrP^C^ into PrP^Sc^. An increase in cholesterol induces PrP^C^ translocation from raft to non-raft regions, protecting from PrP^Sc^-mediated neurodegeneration [58]. In contrast, reduced sphingolipid content supports prion conversion [46]. We tested the effect of modifying the content of gangliosides in CGCs, by incubating cells with exogenous GM3, GM1 or GD1a [3], [25], [32], [59]. The efficiency of ganglioside incorporation in CGCs at 37°C depended on the ganglioside species. Taking into account the endogenous content of each ganglioside in CGC-DRMs [59] (traces of GM3, and 1.1 and 3 nmoles/mg protein of GM1 and GD1a, respectively), administration of exogenous gangliosides resulted in a marked increase of GM3, and in a 3.2 and 1.3 fold increase of GM1 and GD1a. Strikingly, after GM1 enrichment, the PrP^C^ band detected with 6H4Ab was poorly visible in DRMs, whereas no appreciable differences were detected in GM3 and GD1a treated-cells. The distribution of SAF32-immunoreactive PrP^C^ in the gradient fractions did not significantly change after treatment with the gangliosides. Thus increasing ganglioside amount in lipid rafts did not influence PrP^C^ redistribution between raft and not-raft membrane regions.

The results observed are imputable to the added glycolipids, in particular GM1, since only a very limited metabolism of tritiated gangliosides was observed. This effect is concentration-dependent and temperature-dependent. In fact: i) the distribution of Thy1, a GPI-anchored protein particularly enriched in lipid rafts [32], [60], is not subjected to changes in GM1-treated-CGCs, suggesting that PrP^C^/GM1 interaction is specific; ii) the effect of GM1 on PrP^C^ structure is dependent on the amount of ganglioside in lipid rafts. Indeed, our data suggest that PrP^C^/GM1 interaction is “concentration-dependent”; iii) in experiments performed at low temperature (4°C) to decrease the extent of endocytosis [61], [62], the behavior of PrP^C^ in GM1-treated cells is comparable to control CGCs, indicating that the PrP^C^/GM1 interaction is “temperature-dependent”.

Furthermore the strong reduction of PrP^C^ visualized by 6H4Ab in DRMs, in GM1-treated CGCs, is not caused by PrP^C^ cleavage or its redistribution in the sucrose gradient fractions, as shown by WB experiments on deglicosylated protein, allowing the clear visualization of truncated form of PrP^C^, using different antibodies [33], [39]. The results obtained after deglycosylation were also indirectly confirmed by parallel experiments showing no increased recruitment of ADAM10 or ADAM17, known to contribute and regulate PrP^C^ cleavage [12], [13]. Noteworthy, in GM1-treated CGCs, after deglycosylation, 6H4Ab is able to reveal PrP^C^.

Treatment with denaturant facilitates the exposure of cryptic epitopes of PrP^Sc^ [63] and our speculation is that in techniques incorporating a denaturing step 6H4Ab showed good binding indicating increased accessibility of the binding site [55], [64]. Furthermore, in experiments with radiolabeled GM1 radiochromatoscanning of the polyvinylidenedifluoride membrane (PVDF) (data not shown) displayed the absence of radioactivity at 35 kDa, indicating that PrP^C^/GM1 interaction is not denaturation resistant and consequently GM1 does not “mask” the epitope recognized by 6H4Ab.

Sanghera et al. [65] recently, demonstrated that GM1 specifically binds to PrP^C^ and these changes are consistent with a reduction in the amount of random coil structure in the protein. Our CD analysis of recPrP in the presence of GM1-containing liposomes showed a significant loss of α-helical contents with important structural reorganization in PrP^C^ folding. It is known that gangliosides enter in the plasma membrane as monomers using the lipid rafts as “preferential door” [61], [66], [67] and the increase of gangliosides enhances the formation of endocytotic vesicles [68] where, due to the acidic pH, the PrP^C^ preferentially assumes PrP^Sc^-like conformation [69]. Our results reveal that only GM1 ganglioside is able to modify PrP^C^ folding, probably when PrP^C^/GM1 complex are internalized into endocytic compartment from which most of the molecules are recycled intact to the cell surface [53], [60], [70].

### In conclusion, our data showed that native PrP^C^ localize preferentially in DRMs and demonstrated that GM1 ganglioside alter PrP^C^ conformation

We propose that lipid-prion protein interactions on the cell surface can mediate protein function, playing a role in the mechanism underlying prion diseases, thus predisposing to pathology development. As reported by Castilla and Goni [71] the differences in GM1 density might be crucial: a plausible model to explain the convertibility of PrP^C^ in PrP^Sc^ might be depending on the GM1 binding that would slightly modify the PrP^C^ structure, making it more suitable for conversion. The stabilization of PrP^Sc^-like conformation may act as a seed for the further PrP^C^ recruitment and modification, an aspect shared with other neurodegenerative diseases [72]–[74].

## Supporting Information

Figure S1 Immunofluorescence analysis of PrPC localization in the endoplasmic reticulum. Panels A and B: CGCs were double-stained with PrP^C^ 6H4Ab (A, green) and SAF32Ab (B, green) with calreticulin (red) to visualize the endoplasmic reticulum. Scale bar: 10 µm.
(TIF)

Figure S2 Effect of GM3 and GD1a treatment on PrP^C^ distribution. Panel A–D: immunofluorescence analysis of CGCs with anti-PrP^C^ 6H4Ab (A and C), SAF32Ab (B and D) and CTB (red) in the presence of GM3 (A and B) or GD1a (C and D). Ganglioside treatments do not to induce remarkable changes in PrP^C^ distribution. Scale bar: 10 µm.
(TIF)

Figure S3 Effect of gangliosides treatment on ADAM17 localization in CGC gradient fractions. Cells, after incubation with different gangliosides (GM3, GM1 or GD1a) and correspondent radiolabelled gangliosides ([3H]GM3, [3H]GM1 or [3H]GD1a), at a final concentration of 2×10^−6^ M at 37°C for 4 h, were treated with 1% Triton X-100-containing buffer for 30 min on ice. The cellular lysate was subjected to discontinuous sucrose density gradient centrifugation. One-milliliter fractions were withdrawn from the gradient, submitted to 15% SDS-PAGE (20 µg proteins/lane), transferred to nitrocellulose membranes, and immunoblotted with anti-ADAM17 antibody followed by ECL detection. Representative blots from three independent experiments are shown.
(TIF)

Figure S4 Dose (DDt, 10^−6^ M GM1 a 37°C) and temperature (TDt, 2×10^−6^ M GM1 a 4°C) dependence of PrP^C^ distribution in GM1-treated CGCs. Panels A and B: CGCs were double immunolabelled with 6H4Ab (green) and CTB (red) following DDt (A) and TDt (B) treatments. C and D: double staining with SAF32 Ab (green) and CTB (red) following DDt (C) and TDt (D) treatment. Scale bar: 10 µm.
(TIF)

Figure S5 Characterization of PrP^C^ in gradient fractions from control and GM1-treated CGCs. Cells, before and after the incubation with GM1 and correspondent radiolabelled gangliosides [3H]GM1, at a final concentration of 2×10^−6^ M at 37°C for 4 h (Standard treatment, St), were treated with 1% Triton X-100-containing buffer for 30 min on ice. The cellular lysate was submitted to discontinuous sucrose density gradient centrifugation. One-milliliter fractions were withdrawn from the gradient, submitted to 15% SDS-PAGE (20 µg protein/lane), transferred to nitrocellulose membranes and immunoblotted with C20 or 6D11 antibodies against PrP^C^ followed by ECL detection. Representative blots from three independent experiments are shown. C  =  control; GM1  =  GM1-treated CGCs.
(TIF)
