# Supplementary figures and images for: Role of Lipid Rafts and GM1 in the Segregation and Processing of Prion Protein
Source: PLoS One. 2014 May 23;9(5):e98344. doi: 10.1371/journal.pone.0098344 (PMC4032283; doi:10.1371/journal.pone.0098344)

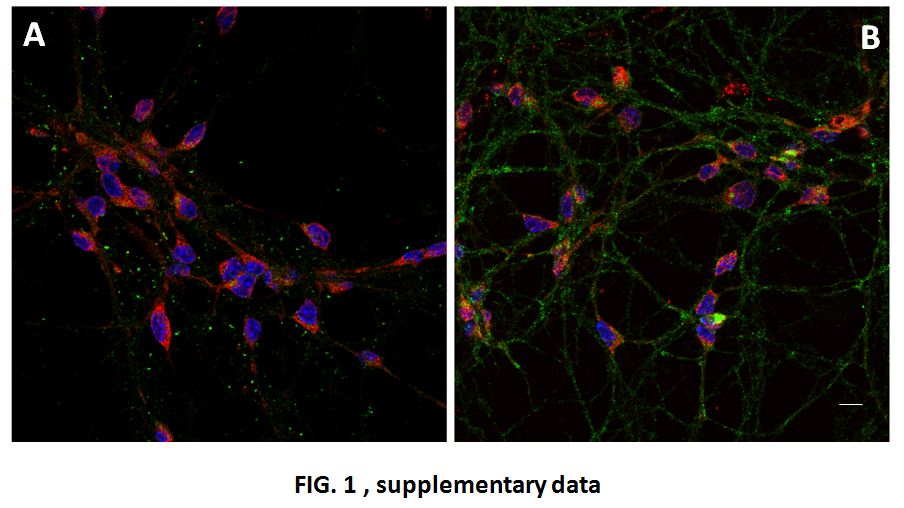

Supplement: Figure S1 — Immunofluorescence analysis of PrPC localization in the endoplasmic reticulum. Panels A and B: CGCs were double-stained with PrPC 6H4Ab (A, green) and SAF32Ab (B, green) with calreticulin (red) to visualize the endoplasmic reticulum. Scale bar: 10 µm. (TIF) [file pone.0098344.s001.tif]

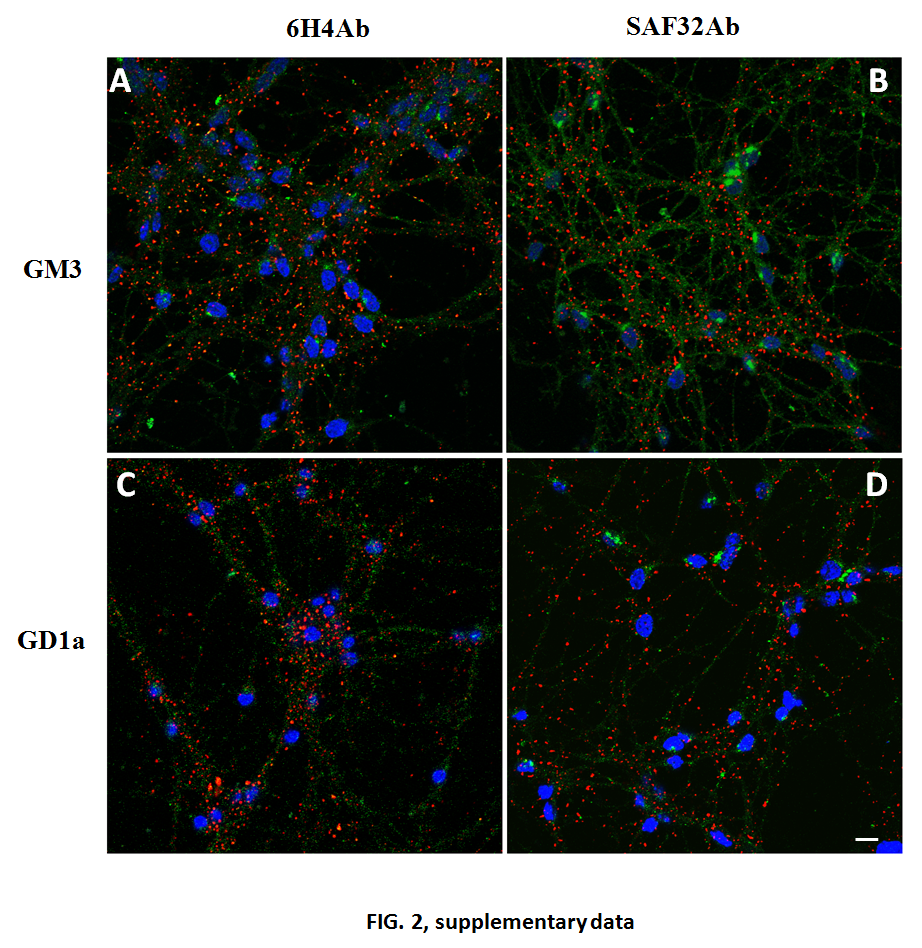

Supplement: Figure S2 — Effect of GM3 and GD1a treatment on PrPC distribution. Panel A–D: immunofluorescence analysis of CGCs with anti-PrPC 6H4Ab (A and C), SAF32Ab (B and D) and CTB (red) in the presence of GM3 (A and B) or GD1a (C and D). Ganglioside treatments do not to induce remarkable changes in PrPC distribution. Scale bar: 10 µm. (TIF) [file pone.0098344.s002.tif]

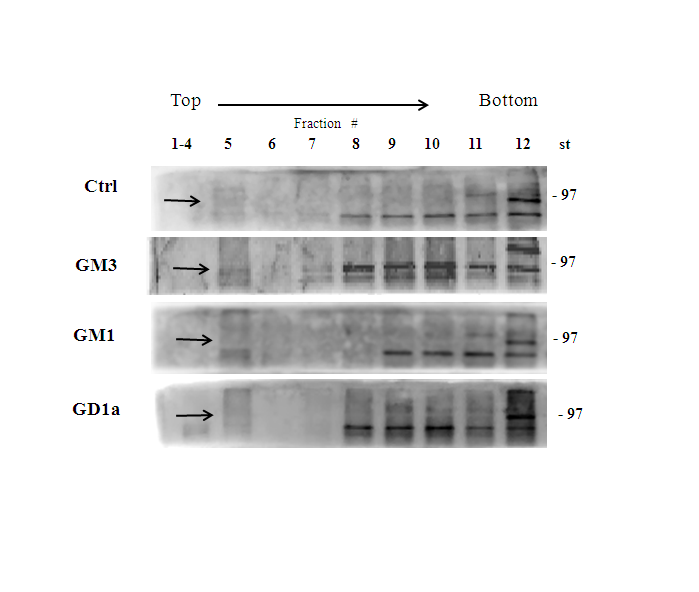

Supplement: Figure S3 — Effect of gangliosides treatment on ADAM17 localization in CGC gradient fractions. Cells, after incubation with different gangliosides (GM3, GM1 or GD1a) and correspondent radiolabelled gangliosides ([3H]GM3, [3H]GM1 or [3H]GD1a), at a final concentration of 2×10^−6^ M at 37°C for 4 h, were treated with 1% Triton X-100-containing buffer for 30 min on ice. The cellular lysate was subjected to discontinuous sucrose density gradient centrifugation. One-milliliter fractions were withdrawn from the gradient, submitted to 15% SDS-PAGE (20 µg proteins/lane), transferred to nitrocellulose membranes, and immunoblotted with anti-ADAM17 antibody followed by ECL detection. Representative blots from three independent experiments are shown. (TIF) [file pone.0098344.s003.tif]

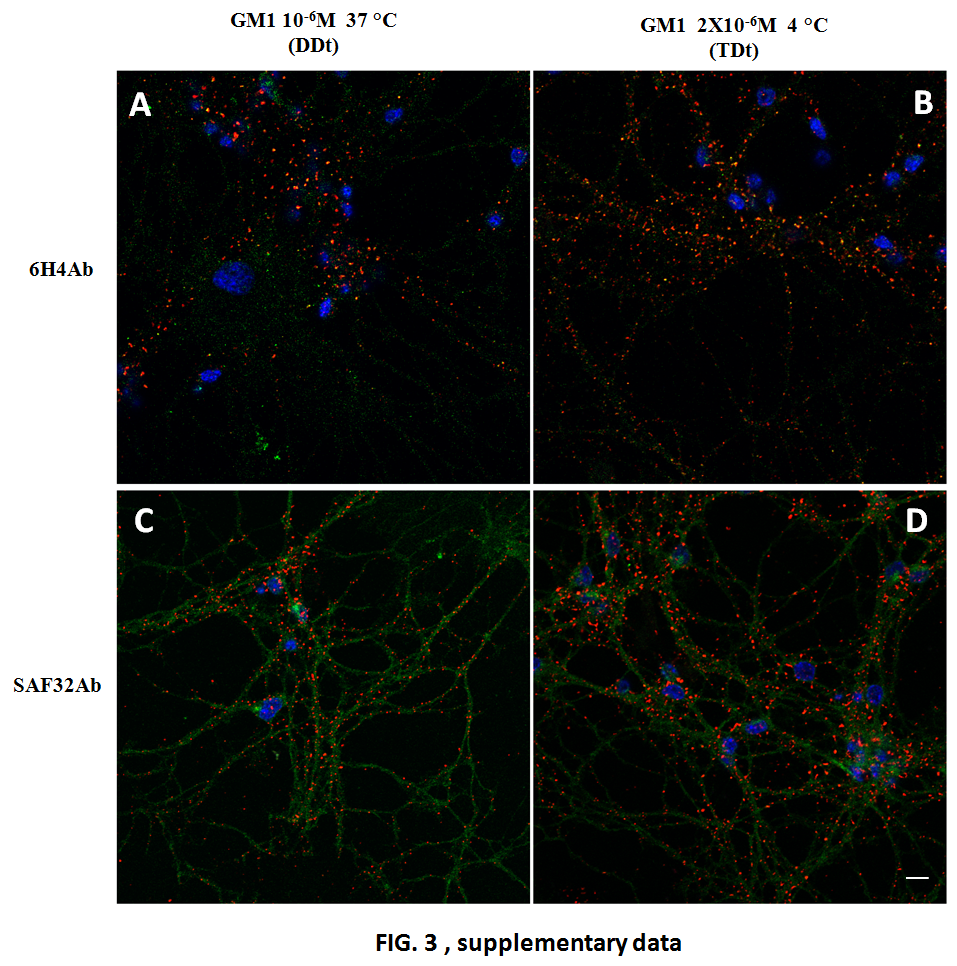

Supplement: Figure S4 — Dose (DDt, 10−6 M GM1 a 37°C) and temperature (TDt, 2×10^−6^ M GM1 a 4°C) dependence of PrPC distribution in GM1-treated CGCs. Panels A and B: CGCs were double immunolabelled with 6H4Ab (green) and CTB (red) following DDt (A) and TDt (B) treatments. C and D: double staining with SAF32 Ab (green) and CTB (red) following DDt (C) and TDt (D) treatment. Scale bar: 10 µm. (TIF) [file pone.0098344.s004.tif]

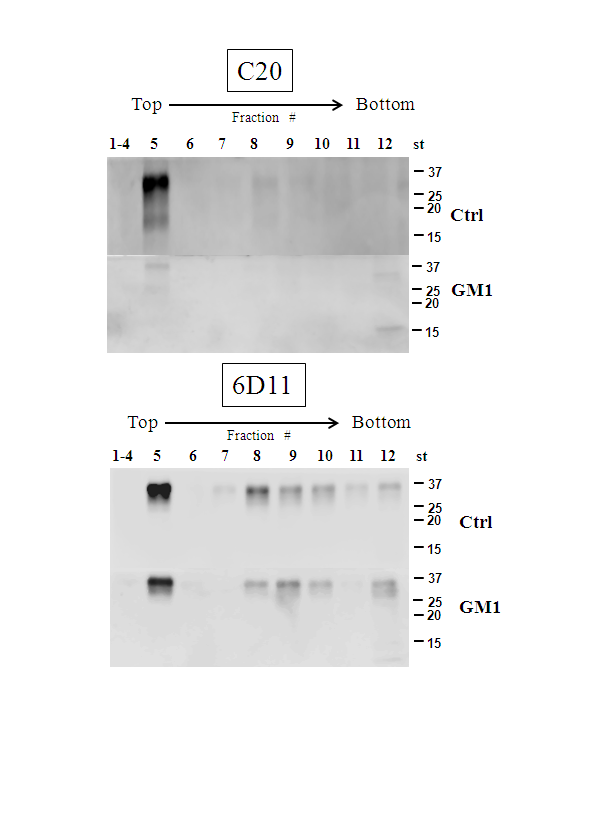

Supplement: Figure S5 — Characterization of PrPC in gradient fractions from control and GM1-treated CGCs. Cells, before and after the incubation with GM1 and correspondent radiolabelled gangliosides [3H]GM1, at a final concentration of 2×10^−6^ M at 37°C for 4 h (Standard treatment, St), were treated with 1% Triton X-100-containing buffer for 30 min on ice. The cellular lysate was submitted to discontinuous sucrose density gradient centrifugation. One-milliliter fractions were withdrawn from the gradient, submitted to 15% SDS-PAGE (20 µg protein/lane), transferred to nitrocellulose membranes and immunoblotted with C20 or 6D11 antibodies against PrPC followed by ECL detection. Representative blots from three independent experiments are shown. C = control; GM1 = GM1-treated CGCs. (TIF) [file pone.0098344.s005.tif]
